# The pervasive effects of recombinant *Fasciola gigantica* Ras-related protein Rab10 on the functions of goat peripheral blood mononuclear cells

**DOI:** 10.1186/s13071-018-3148-2

**Published:** 2018-11-06

**Authors:** Ai-Ling Tian, MingMin Lu, Fu-Kai Zhang, Guillermo Calderón-Mantilla, Evangelia Petsalaki, XiaoWei Tian, WenJuan Wang, Si-Yang Huang, XiangRui Li, Hany M. Elsheikha, Xing-Quan Zhu

**Affiliations:** 1 State Key Laboratory of Veterinary Etiological Biology, Key Laboratory of Veterinary Parasitology of Gansu Province, Lanzhou Veterinary Research Institute, Chinese Academy of Agricultural Sciences, Lanzhou, Gansu Province 730046 People’s Republic of China; 2 College of Veterinary Medicine, Nanjing Agricultural University, Nanjing, 210095 People’s Republic of China; 3 European Molecular Biology Laboratory-European Bioinformatics Institute, Wellcome Genome Campus, Hinxton, CB10 1SD UK; 4 College of Veterinary Medicine, Yangzhou University, Yangzhou, Jiangsu Province 225009 People’s Republic of China; 5 Jiangsu Co-innovation Center for the Prevention and Control of Important Animal Infectious Diseases and Zoonoses, Yangzhou, Jiangsu Province 225009 People’s Republic of China; 6 Faculty of Medicine and Health Sciences, School of Veterinary Medicine and Science, University of Nottingham, Sutton Bonington Campus, Loughborough, LE12 5RD UK

**Keywords:** *Fasciola gigantica*, Ras-related protein Rab10, Recombinant proteins, Peripheral blood mononuclear cells, Immunomodulation

## Abstract

**Background:**

*Fasciola gigantica*-induced immunomodulation is a major hurdle faced by the host for controlling infection. Here, we elucidated the role of *F. gigantica* Ras-related protein Rab10 (FgRab10) in the modulation of key functions of peripheral blood mononuclear cells (PBMCs) of goats.

**Methods:**

We cloned and expressed recombinant FgRab10 (rFgRab10) protein and examined its effects on several functions of goat PBMCs. Protein interactors of rFgRab10 were predicted *in silico* by querying the databases Intact, String, BioPlex and BioGrid. In addition, a total energy analysis of each of the identified interactions was also conducted. Gene Ontology (GO) enrichment analysis was carried out using FuncAssociate 3.0.

**Results:**

The *FgRab10* gene (618 bp), encodes 205-amino-acid residues with a molecular mass of ~23 kDa, had complete nucleotide sequence homology with *F. hepatica* Ras family protein gene (PIS87503.1). The rFgRab10 protein specifically cross-reacted with anti-*Fasciola* antibodies as shown by Western blot and immunofluorescence analysis. This protein exhibited multiple effects on goat PBMCs, including increased production of cytokines [interleukin-2 (IL-2), IL-4, IL-10, transforming growth factor beta (TGF-β) and interferon gamma (IFN-γ)] and total nitric oxide (NO), enhancing apoptosis and migration of PBMCs, and promoting the phagocytic ability of monocytes. However, it significantly inhibited cell proliferation. Homology modelling revealed 63% identity between rFgRab10 and human Rab10 protein (Uniprot ID: P61026). Protein interaction network analysis revealed more stabilizing interactions between Rab proteins geranylgeranyltransferase component A 1 (CHM) and Rab proteins geranylgeranyltransferase component A 2 (CHML) and rFgRab10 protein. Gene Ontology analysis identified RabGTPase mediated signaling as the most represented pathway.

**Conclusions:**

rFgRab10 protein exerts profound influences on various functions of goat PBMCs. This finding may help explain why *F. gigantica* is capable of provoking recognition by host immune cells, less capable of destroying this successful parasite.

**Electronic supplementary material:**

The online version of this article (10.1186/s13071-018-3148-2) contains supplementary material, which is available to authorized users.

## Background

*Fasciola gigantica* and *Fasciola hepatica* are omnipresent agents of a zoonotic parasitic disease, fascioliasis, which continues to be a major health burden on animals and humans. Fascioliasis can adversely affect the sustainability of the farm animal industry [1]. The annual global economic loss due to fascioliasis has been estimated to be in excess of three billion dollars [2]. Worldwide, at least 2.4 million people have been infected with fascioliasis, with a further 180 million people at risk of being infected [3]. Despite this high impact and investigations for decades using clinical studies as well as animal models, knowledge about host defense mechanisms against *F. gigantica* is limited. This challenge is partly due to the fact that *Fasciola* spp. are very efficient modulators of the host immune response [4]. The immunomodulatory capacity of *F. gigantica*, mediated by parasite-derived effector molecules, is believed to play important roles in the establishment of long-lasting infection in the host.

Several studies have investigated various excretory/secretory products (ESPs) and virulence effector molecules employed by *F. gigantica* flukes to ensure their survival and establishment of persistent infection [5, 6]. Rab proteins are a family of small GTP-binding proteins, part of the Ras superfamily, which regulate intracellular membrane trafficking of several pathogens; including parasites (e.g. *Plasmodium*, *Theileria*, *Cryptosporidium* and *Babesia* [7] and *Toxoplasma gondii* [8]), bacteria (e.g. *Mycobacterium* spp. [9] and *Listeria monocytogenes* [10]) and fungi [11]. Despite their crucial role as regulators of vesicular membrane traffic, the roles of Rab proteins in the pathogenesis of *F. gigantica* infection remain largely unknown. Understanding the influence of parasite-secreted proteins on the function of immune cells, such as goat peripheral blood mononuclear cells (PBMCs), is essential due to their important role in the immunopathogenesis of fascioliasis [12]. In a recent study, we cloned and expressed a recombinant *F. gigantica* 14-3-3 epsilon protein (rFg14-3-3e), and characterized its effects on specific functions of goat PBMCs [6].

In the present study, we expand our investigation of the effects of *F. gigantica* ESPs on the functions of these immune cells. Specifically, the gene encoding *F. gigantica* Rab10 (FgRab10) was cloned and expressed in *Escherichia coli*. Then, the modulatory effects of the purified recombinant FgRab10 (rFgRab10) protein on the functions of goat PBMCs; including cytokine secretion, proliferation, migration, nitric oxide (NO) production, phagocytosis and apoptosis were investigated. Our results indicate that rFgRab10 protein can significantly influence key functions of goat PBMCs, all are critical facets of the immunopathogenesis of *F. gigantica* infection.

## Methods

### Animals

Three crossbred goats (3–6 months-old) were obtained from the teaching and research flock at Nanjing Agricultural University. Goats were treated with triclabendazole (50 mg/kg body weight) in order to exclude the possibility of any prior infection with liver flukes. Two weeks post-treatment, a faecal specimen from each goat was examined microscopically to exclude the presence of helminth eggs. Female Sprague Dawley (SD) rats (150–200 g) were purchased from the Experimental Animal Center of Jiangsu Province, China (Certificate: SCXK 2008-0004), and used for the production of antibodies. Rats were raised under specific pathogen-free conditions, and fed with sterilized food and water *ad libitum*. All efforts were made to minimize the suffering of animals, and daily health checks were performed throughout the experiment.

### Purification and culture of goat PBMCs

Venous blood samples were collected from the jugular vein of goats, with no history of *F. gigantica* infection or other parasitic infections, into Vacutainer tubes coated with ethylenediaminetetraacetic acid (EDTA). PBMCs were isolated from freshly collected blood using a PBMC isolation kit (TBD, Tianjin, China). Culturing was done by incubating the isolated PBMCs in RPMI 1640 medium containing 10% fetal bovine serum (FBS) and 1% penicillin-streptomycin (Gibco, New York, USA). Cultures were maintained in a humidified atmosphere of 5% CO_2_ at 37 °C. The number of PBMCs was adjusted to 10^6^ cells/ml in RPMI 1640 medium and cell viability was assessed using trypan blue dye exclusion method. The number of viable cells was counted in a haemocytometer and only cells with > 95% viability were used in the experiments. All assays were performed using freshly isolated PBMCs. For the monocyte phagocytosis experiment, adherent monocytes were obtained after incubation of PBMCs for 48 h at 37 °C in 5% CO_2_, followed by removal of the non-adherent cells. In all assays performed to investigate the effects of rFgRab10 protein on certain functions of goat PBMCs and monocytes, cultured cells were exposed to rFgRab10 protein at concentrations of 10 μg/ml, 20 μg/ml, 40 μg/ml or 80 μg/ml. PBMCs treated with SUMO protein expressed in pET-SUMO expression vector and sham-treated with phosphate buffered saline (PBS, pH 7.4) were used as controls in all assays. All experiments were performed in triplicate.

### Parasite strain

Adult *F. gigantica* flukes were harvested from the gall-bladder of naturally infected buffaloes at local abattoirs in Guangxi Zhuang Autonomous Region, PR China. The collected flukes were washed twice in PBS and used for RNA isolation or stored frozen at -80 °C with RNA stabilizer for later analysis. The identity of the flukes collected was ascertained to be *F. gigantica* based on PCR and sequencing analysis of the second internal transcribed spacer (ITS2) of ribosomal DNA [13]. This analysis revealed 100% ITS2 sequence homology between the flukes collected and *F. gigantica* samples examined previously from the same region (GenBank: AJ557569).

### Cloning and characterization of *FgRab10* gene

Because *F. gigantica* genome sequences are not available, we searched the NCBI/BLASTx (https://blast.ncbi.nlm.nih.gov/Blast.cgi), *F. hepatica* cDNA library and liquid chromatography-tandem mass spectrometry (LC-MS/MS)-based *F. hepatica* ESPs dataset from previously unpublished proteomics studies to identify homologous Ras family protein gene sequences. This analysis identified *F. hepatica Ras* family protein gene (GenBank: PIS87503.1), which was utilized to design primers to amplify *F. gigantica* Ras-related protein *Rab10* (*FgRab10*) gene. Total RNA was isolated from adult *F*. *gigantica* flukes using Trizol reagent (Invitrogen, San Diego, USA), and was used to synthesize first-strand cDNA with reverse transcription polymerase chain reaction (RT-PCR) using the RevertAid First Strand cDNA Synthesis Kit [Thermo Scientific (EU), Vilnius, Lithuania]. The cDNA was used for the amplification of *FgRab10* gene using a forward primer (5'-CCG GAA TTC ATG GCT AAG AAG TCG TAC GAT G -3') and a reverse primer (5'-ATT TGC GGC CGC TGT AGG ACA CCA GGA GCA-3'). *Eco*R I and *Not* I restriction sites, which were underlined, were incorporated into the primers. The amplified *FgRab10* gene was digested with *Eco*R I and *Not* I, and ligated into the corresponding cloning sites in the T-vector pMD19 (Simple) (Takara, Dalian, Liaoning, China). The recombinant plasmid was transformed into *Trans5α* chemically competent cells (TransGen Biotech, Beijing, China). Positive clones were sequenced by GenScript (Nanjing, Jiangsu, China) in order to confirm the correct insertion/orientation of *FgRab10* gene in the vector. The *FgRab10* cDNA sequence was translated into amino acid sequences using EditSeq (DNASTAR Lasergene). The signal peptide and conserved domains of FgRab10 were predicted by SignalP 4.1 Server (http://www.cbs.dtu.dk/services/SignalP/), and (https://www.ncbi.nlm.nih.gov/Structure/cdd/wrpsb.cgi), respectively.

### Expression and purification of rFgRab10 protein

Positive clones containing the *FgRab10* gene were selected. The *FgRab10* gene fragment was sub-cloned into the pET-SUMO expression vector. The pET-SUMO-*FgRab10* plasmid was transformed into *E. coli* BL2 (DE3), then induced by addition of 1 mM isopropyl-β-d-thiogalactopyranoside (IPTG) (Solarbio, Beijing, China) to a culture with an optical density at 600 nm (OD_600_) of 0.6 at 37 °C. After 5 h, the bacterial cells were collected by centrifugation (10,000× *g*, 10 min, 4 °C), resuspended in 5 ml imidazole buffer (20 mM phosphate, 0.5 M NaCl, 10 mM imidazole), and frozen at -20 °C. The cell preparation was sonicated at 600 W for 20 min and further centrifuged at 10,000× *g* for 10 min at 4 °C, and the recombinant His-tagged protein was isolated from bacterial pellets under denaturing conditions in a solution containing 8 M urea. After a third centrifugation, the supernatant was purified using the His GaviTrap kit (GE Healthcare, Buckinghamshire, UK). The eluted protein was first dialyzed in a solution containing 0.1 M urea to be refolded and renatured in a buffer containing a linear decreased urea gradient (6 M, 4 M, 2 M and 0 M urea), and then dialyzed against PBS to remove imidazole. The non-recombinant pET-SUMO expression vector (without *FgRab10* gene) was used to produce the control His-tagged SUMO protein, which was expressed and purified using the same method used to obtain rFgRab10 protein. The lipopolysaccharide (LPS) was depleted from the rFgRab10 protein using Detoxi-Gel Affinity Pak prepacked columns (Thermo Scientific, Waltham, MA, USA). The concentration of the rFgRab10 protein was adjusted to 1 mg/ml. The endotoxin level of the sample was measured with a Limulus Amebocyte Lysate (LAL) gel clot assay using a Pyrosate® Kit (Cape Cod Inc., East Falmouth, MA, USA).

### Preparation of polyclonal antibodies

Polyclonal antibodies were produced by subcutaneous immunization of SD rats with purified rFgRab10 protein. Briefly, SD rats were immunized with 300 μg of the purified protein emulsified with complete Freund’s adjuvant (1:1). After each 2-week interval, SD rats were boosted four times with the same dose of the rFgRab10 protein mixed with incomplete Freund’s adjuvant. One week after the last injection, rat serum was collected and stored frozen at -80 °C.

### Western blot analysis of rFgRab10 protein

The purity of the protein was detected by 12% SDS-PAGE, followed by Coomassie blue staining. Also, 20 μg of the purified rFgRab10 protein were resolved on 12% SDS-PAGE and transferred onto Hybond-C extra nitrocellulose membrane (Amersham, London, UK). The membrane was washed 5 times (5 min each) in TBS-T. The membrane was then incubated with primary antibodies (serum from sheep naturally infected with *Fasciola*) for 1 h at 37 °C (1:100 in TBS-T). After washing 5 times in TBS-T, the membrane was incubated with horseradish peroxidase (HRP)-conjugated rabbit anti-goat immunoglobulin (Ig)-G antibody (Sigma, St. Louis, MO, USA) at 37 °C for 1 h (1:2000 in TBS-T). The immunoreaction was visualized using WesternBright ECL (Advansta, Menlo Park, CA, USA) and LucentBlue X-ray film (Advansta, Menlo Park, CA, USA).

### Immunofluorescence staining of rFgRab10 protein bound to goat PBMCs

PBMCs were incubated with rFgRab10 protein in a humidified atmosphere of 5% CO_2_ at 37 °C for 1 h. Cells were fixed with 4% paraformaldehyde, washed 3 times in PBS and treated with blocking solution (4% BSA in PBS) for 1 h to minimize background staining. rFgRab10-treated, SUMO protein-treated, or PBS-treated PBMCs were incubated with rat anti-rFgRab10 protein antibody (1:100 in 4% BSA) for 1 h at 37 °C. Cells were stained with Cy3 conjugated goat anti-rat IgG secondary antibody (1:500 in 4% BSA) (Beyotime, Jiangsu, China) for 1 h at 37 °C. Hoechst 33342 (Invitrogen, Oregon, USA) was used to stain the nuclei. Stained cells were imaged using a confocal laser scanning microscope (LSM710, Zeiss, Jena, Germany) and digital images were analyzed using Zen 2012 imaging software.

### Quantification of cytokines in goat PBMCs culture supernatant

Cytokine production was detected in the culture supernatants of PBMCs incubated with rFgRab10 protein. PBMCs were plated at a cell density of 10^6^ cells/well in 24-well tissue culture plates in 1 ml culture medium with various concentrations (10, 20, 40 and 80 μg/ml) of rFgRab10 protein. The culture plates were maintained in 5% CO_2_ at 37 °C for 24 h. The PBMCs supernatant was then collected and assayed for the production of interleukin (IL)-2, IL-4, IL-10, transforming growth factor beta (TGF-β) and interferon gamma (IFN-γ) using goat ELISA kit (Mlbio, Shanghai, China). Limits of detection were between 2 and 1200 pg/ml depending on the cytokine.

### Assessment of nitric oxide (NO) production

PBMCs were seeded into 24-well flat-bottomed plates at 10^6^ cells per well in 1 ml RPMI medium. PBMCs were allowed to adhere for 2 h in 5% CO_2_ at 37 °C; cells were then stimulated by rFgRab10 protein using the same concentrations used above. The supernatant was collected after 24 h and analyzed for NO production by Total Nitric Oxide Assay Kit (Beyotime, Haimen, Jiangsu, China). A microplate reader (Bio-Rad, Hercules, CA, USA) was used to measure the optical density at 540 nm (OD_540_). NO levels were calculated against a standard curve generated using 0-80 μM/l sodium nitrites.

### Determination of apoptosis assay using Annexin V/PI staining

PBMCs were seeded into 24-well tissue culture plates at 10^6^ cells/well in 1 ml RPMI medium with each of the rFgRab10 protein concentrations used above. The plate was incubated in 5% CO_2_ at 37 °C for 24 h. PBMCs were then stained with Annexin V and PI using the Annexin V-FITC kit (Miltenyi Biotec, Bergisch Gladbach, Nordrhein-Westfalen, Germany). Apoptosis in PBMCs was quantified by flow cytometry. Data were analyzed using FlowJo 10. Annexin V-positive/PI-negative PBMCs were considered to be apoptotic and were analyzed as a percentage of the entire PBMCs population.

### *In vitro* goat PBMCs migration assay

To test whether rFgRab10 protein can stimulate the *in vitro* the migration of PBMCs, we assessed the movement of untreated and rFgRab10-tretaed PBMCs from the upper chamber of a Transwell plate (Corning, Kennebunk, USA), through an 8-μm-pore polycarbonate membrane inserts toward the lower chamber. Briefly, ~10^6^ cells in 100 μl RPMI medium without or with rFgRab10 at the concentrations used above were seeded into the upper chamber of the Transwell in 24-well tissue culture plates. The bottom chamber was filled with ~600 μl RPMI medium. The plate was incubated in 5% CO_2_ at 37 °C for 4 h. After incubation, PBMCs were collected from the bottom chamber and the number of cells migrated through the membrane into the lower chamber was determined using an Automated Cell Counter (Countstar, Shanghai, China). The results are presented as percentages of the seeded PBMCs.

### Measurement of FITC-dextran uptake

Goat monocytes were seeded into 24-well tissue culture plates at 10^6^ cells/well in 1 ml RPMI medium with the various rFgRab10 protein concentrations. The plate was incubated in 5% CO_2_ at 37 °C for 48 h. After centrifugation at 500× *g*, monocytes were resuspended in chilled PBS, and incubated with 1 mg/ml FITC-dextran (average molecular weight of dextran, 4000; Sigma, St. Louis, MO, USA) in RPMI 1640 Medium at 4 °C or 37 °C for 1 h in darkness. To halt phagocytosis, chilled PBS containing 2% FBS was added after incubation and goat monocytes were washed trice with PBS and resuspended in chilled PBS. FITC-dextran uptake of monocytes was analyzed using flow cytometry (BD Biosciences, San Jose, CA, USA). The data were analyzed using FlowJo 10.

### Determination of goat PBMCs proliferation

PBMCs were plated in 96-well tissue culture plates at a cell density of 10^4^ PBMCs/well in triplicate in 100 μl complete RPMI medium with each of the rFgRab10 protein concentrations used above. The plate was incubated in a humidified atmosphere of 5% CO_2_ at 37 °C. After 48 h, 10 μl of Cell Counting Kit-8 (CCK-8) (Beyotime, Jiangsu, China) reagent was added to each well and the plate was further incubated under the same conditions in darkness. A microplate reader (Bio-Rad) was used to measure the optical density at 450 nm (OD_450_).

### Bioinformatics

We used blastx (https://blast.ncbi.nlm.nih.gov/) to obtain the protein sequence of rFgRab10 and the Fragments Library of ROBETTA (http://robetta.bakerlab.org/) [14] to obtain the 3-mer and 9-mer fragments. Using the sequence of rFgRab10, and the 3-mers and 9-mers, the *de novo* 3D protein structure was predicted using ROSETTA’s AbinitioRelax function (https://www.rosettacommons.org/) [15]. The pdb result was aligned to the human Rab10 chain A protein using the Match-maker and match-align tools of UCSF Chimera (https://www.cgl.ucsf.edu/chimera/) [16]. UCSF Chimera was also used to visualize the *ab initio* structure and the alignment to the human Rab10 chain A protein.

Protein Blast [17] was used to retrieve the protein sequence of humans (taxid: 9606), most similar to the rFgRab10 protein sequence. The human Rab10 protein (Uniprot ID: P61026) was used to query interactions in the following databases: Intact [18], String [19], BioPlex [20] and BioGrid [21]. The results were summarized and filtered by P61026 (Rab10) protein using a Python script; this list of interacting pairs was the input used to obtain structure interactions using Interactome3D [22]. All experimental and modeled interactions were used to build a model using the aligned fasta recombinant Rab10 protein sequence to the P61026 (Rab10) protein. A total energy analysis was performed using FoldX Suite [23] in order to calculate the structure energy of each protein involved in the interaction. We used Cytoscape (http://www.cytoscape.org/) to build a protein interaction network. Finally, Gene Ontology (GO) enrichment analysis was performed, using as input the 13 human proteins that interacted with P61026 (Rab10), using FuncAssociate 3.0 [24].

### Statistical analysis

Data values were expressed as the mean ± standard deviation (SD). Differences between the groups were examined for statistical significance using GraphPad Prism 6.0. Statistical analysis was performed using one-way analysis of variance (ANOVA). *P* values < 0.05, 0.01, 0.001 and 0.0001 were represented as *, **, *** and ****, respectively, in comparison with untreated controls. All experiments were repeated at least three independent times.

## Results

### Cloning of *FgRab10* gene

PCR products (~618 bp) of *FgRab10* gene were successfully cloned into T-vector pMD19. The identity of the construct (pMD19T-*FgRab10*) was verified by DNA sequence analysis. FgRab10 is a 205 amino-acid protein with a molecular mass of ~23 kDa (GenBank: MH532439). Conserved domain analysis showed that *FgRab10* protein belonged to P-loop NTPase domain superfamily. Amino acid sequence comparison of the *FgRab10* gene with that of *F. hepatica Ras* family protein gene (GenBank: PIS87503.1) showed 100% sequence homology.

### SDS-PAGE and Western blot analysis

**Fig. 1:**
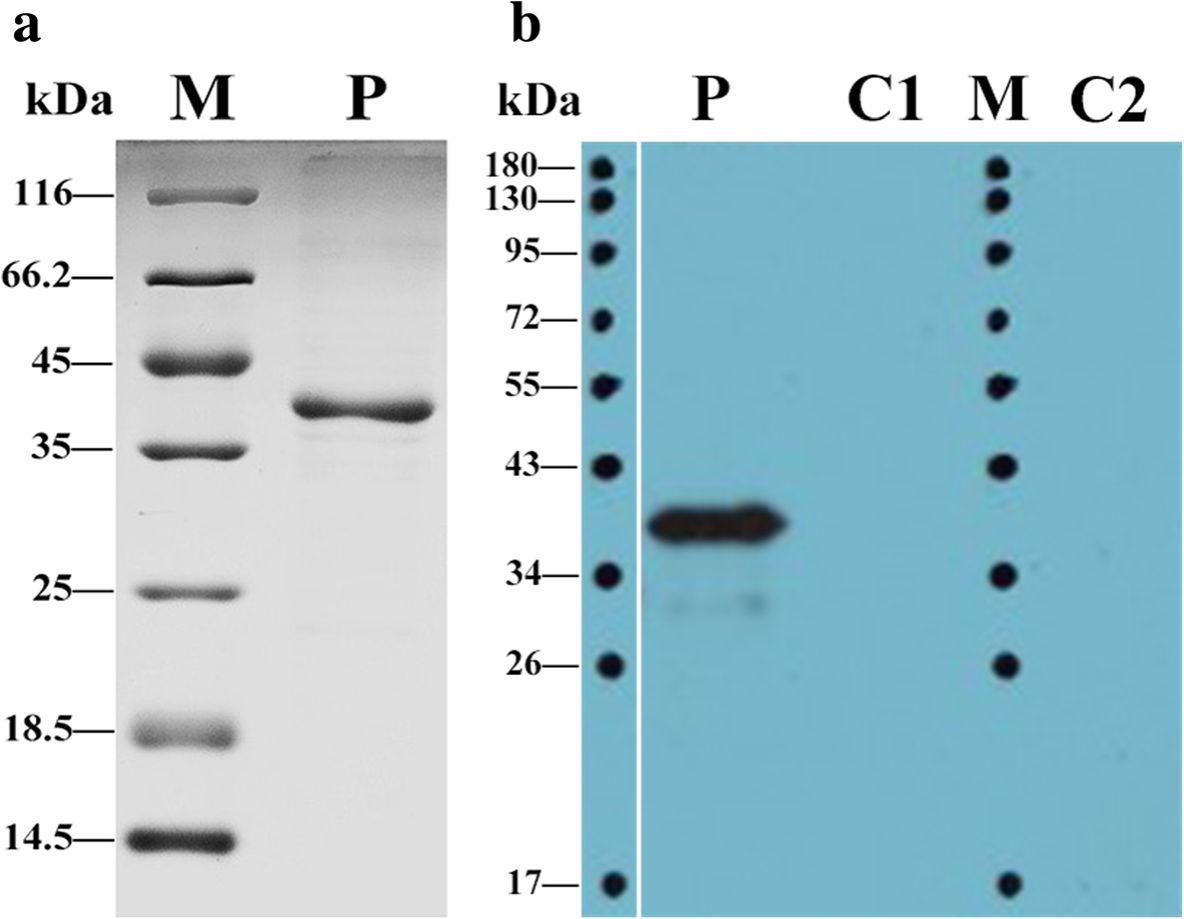
SDS-PAGE and Western blot analyses of the purified rFgRab10 protein from the sonicated culture sediment of *E. coli*. **a** rFgRab10 protein was resolved on 12% acrylamide gel and stained with Coomassie blue. Lane M: prestained protein ladder in kilodaltons; Lane P: purified recombinant protein, rFgRab10, which appeared as a single band of ~39 kDa. **b** rFgRab10 protein was run under non-reducing conditions, and the immunoreaction was visualized using chemiluminescent HRP substrate and X-ray film. Lane M: prestained protein ladder; Lane P: loaded with rFgRab10 protein expressed from *E. coli*. Serum from *Fasciola*-infected sheep detected a single band of ~39 kDa; Lane C1: loaded with protein expressed from sonicated culture supernatant of *E. coli* transformed with empty pET-SUMO vector without *rFgRab10* gene insert, which did not react with goat serum containing anti-*F. gigantica* IgG antibodies; Lane C2: loaded with rFgRab-10 protein expressed from *E. coli* that did not react with the serum of healthy uninfected goats

The *FgRab10* gene fragment was successfully sub-cloned into pET-SUMO expression vector and the positive clones, pET-SUMO-*FgRab10*, were induced by IPTG in *E. coli* BL2 (DE3). The recombinant rFgRab10 protein was successfully expressed as a soluble His-tagged fusion protein using the small ubiquitin-like modifier (SUMO). A protein with an apparent molecular mass of ~39 kDa (~23 kDa rFgRab10 protein + ~16 kDa SUMO tag protein) was detected in SDS-PAGE (Fig. 1a). The specificity of rFgRab10 protein was confirmed by Western blot analysis, wherein rFgRab10 protein specifically reacted with serum from *Fasciola*-infected sheep as indicated by the presence of a single ~39 kDa band. No bands were observed in negative controls [empty pET-SUMO vector without *rFgRab10* gene insert or in samples probed with serum from healthy sheep (Fig. 1b)].

### Binding affinity of rFgRab10 protein to goat PBMCs

**Fig. 2:**
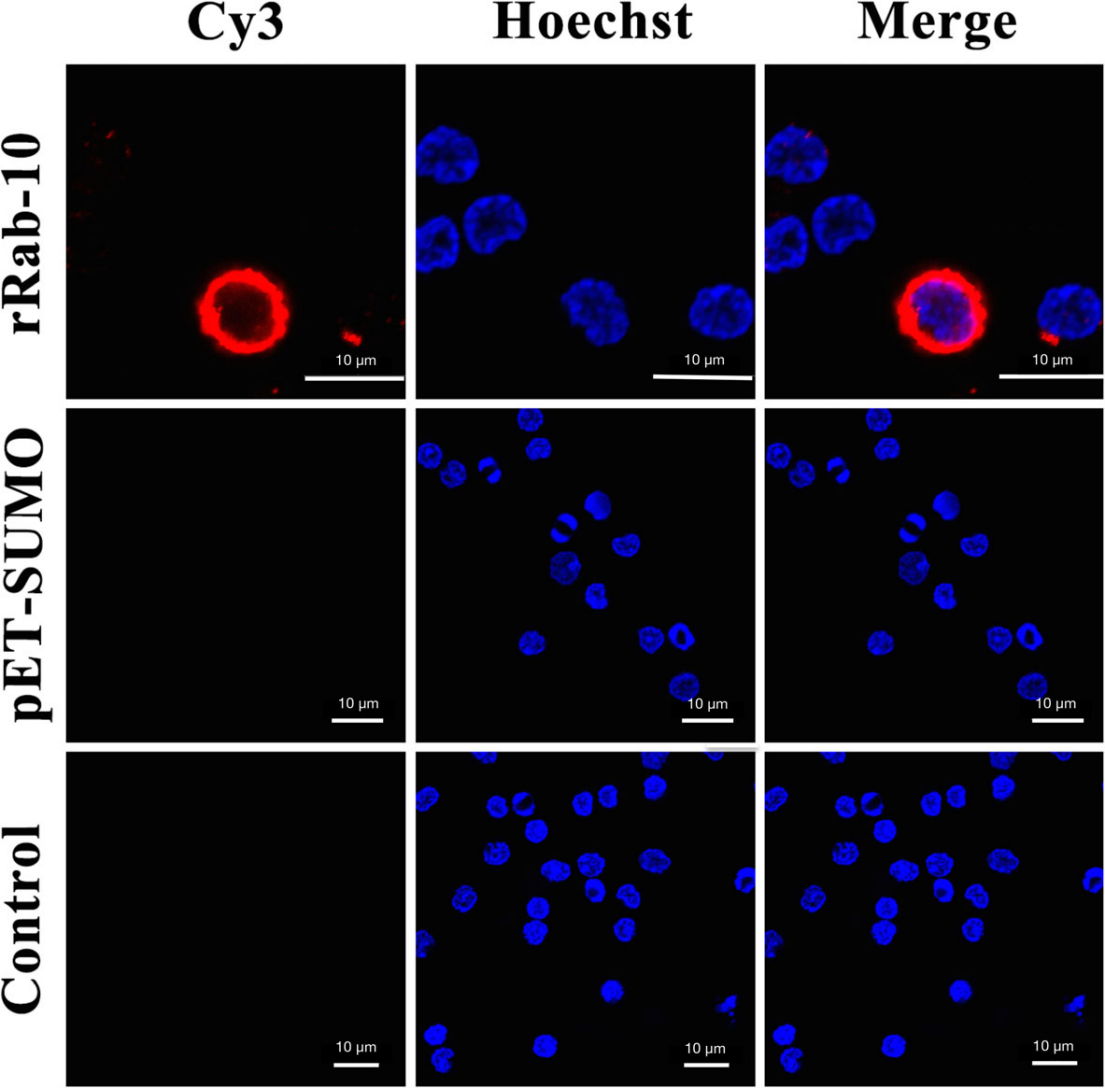
Localization of *F. gigantica*-derived rFgRab10 protein to the PBMCs surface. rFgRab10 protein binding to goat PBMCs surface was determined by incubating PBMCs treated with purified rFgRab10 protein, protein from sonicated culture of *E. coli* transformed with empty pET-SUMO vector or sham-treated with PBS (control) with primary anti-rFgRab-10 protein antibody raised in rats. PBMCs’ nuclei and rFgRab10 protein were stained by Hoechst 33342 (blue) and Cy3-conjugated secondary antibody (red), respectively. Surface staining was detected in rFgRab10-treated cells, but not in PBMCs incubated with SUMO protein and sham-treated PBMCs. *Scale-bars*: 10 μm

Binding of rFgRab10 protein to PBMCs surface was determined by immunofluorescent staining. By incubating rFgRab10-treated PBMCs with specific anti-rFgRab10 antibodies, we detected an even distribution and localization of the red Cy3 dye on the PBMCs membrane, indicating the successful binding of rFgRab10 protein to PBMCs surface (Fig. 2). No fluorescence was observed in PBMCs incubated with SUMO protein obtained from sonicated culture supernatant of *E. coli* transformed with empty pET-SUMO vector or in PBS-treated goat PBMCs.

### rFgRab10 protein modulates cytokine production by goat PBMCs

**Fig. 3:**
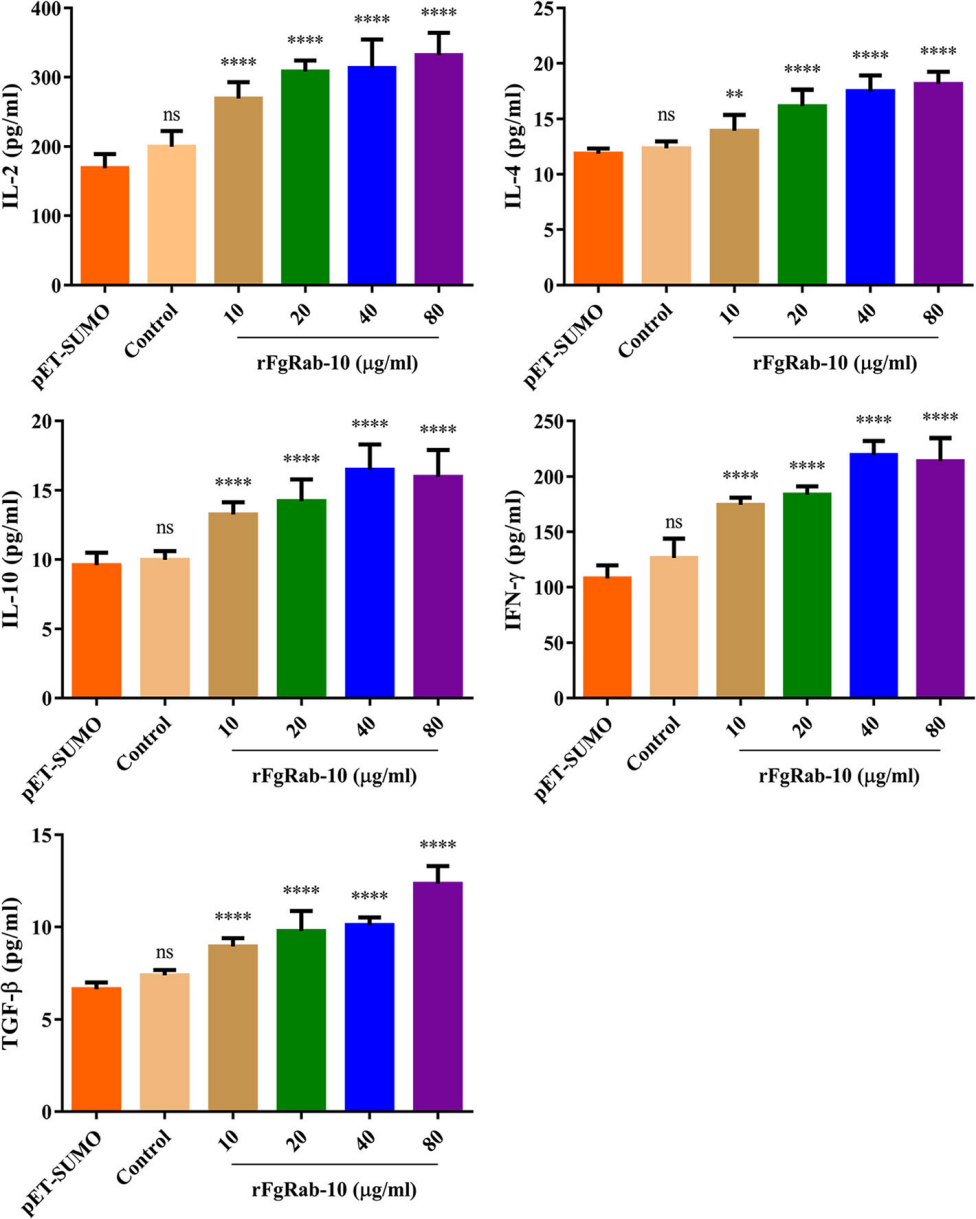
rFgRab10 protein induced the cytokine secretion. Goat PBMCs were incubated for 24 h in the presence of serial concentrations of rFgRab10 protein. PBMCs incubated with SUMO protein or PBS-treated PBMCs served as controls. The levels of cytokine concentration in the supernatant of cultured PBMCs was quantified by ELISA. Graphs represent means ± SD of data from three independent biological replicates. Asterisks indicate statistical significance between the different indicated groups (**P < 0.01; ****P < 0.0001; ns, non-significant)

rFgRab10 protein caused alteration in the levels of five cytokines, IL-2, IL-4, IL-10, IFN-γ and TGF-β. As shown in Fig. 3, when PBMCs were treated with different concentrations of rFgRab10 protein, the production of cytokines was significantly increased, when compared with PBMCs treated with SUMO protein obtained from sonicated culture of *E. coli* transformed with empty pET-SUMO vector (ANOVA tests: IL-2 (0 μg/ml: *F*_(5, 48)_ = 53.63, *P* = 0.0778; 10 μg/ml: *F*_(5, 48)_ = 53.63, *P* < 0.0001; 20 μg/ml: *F*_(5, 48)_ = 53.63, *P* < 0.0001; 40 μg/ml: *F*_(5, 48)_ = 53.63, *P* < 0.0001; 80 μg/ml: *F*_(5, 48)_ = 53.63, *P* < 0.0001), IL-4 (0 μg/ml: *F*_(5, 48)_ = 47.06, *P* = 0.8419; 10 μg/ml: *F*_(5, 48)_ = 47.06, *P* = 0.0020; 20 μg/ml: *F*_(5, 48)_ = 47.06, *P* < 0.0001; 40 μg/ml: *F*_(5, 48)_ = 47.06, *P* < 0.0001; 80 μg/ml: *F*_(5, 48)_ = 47.06, *P* < 0.0001), IL-10 (0 μg/ml: *F*_(5, 48)_ = 39.92, *P* = 0.9661; 10 μg/ml: *F*_(5, 48)_ = 39.92, *P* < 0.0001; 20 μg/ml: *F*_(5, 48)_ = 39.92, *P* < 0.0001; 40 μg/ml: *F*_(5, 48)_ = 39.92, *P* < 0.0001; 80 μg/ml: *F*_(5, 48)_ = 39.92, *P* < 0.0001), TGF-β (0 μg/ml: *F*_(5, 48)_ = 83.54, *P* = 0.0839; 10 μg/ml: *F*_(5, 48)_ = 83.54, *P* < 0.0001; 20 μg/ml: *F*_(5, 48)_ = 83.54, *P* < 0.0001; 40 μg/ml: *F*_(5, 48)_ = 83.54, *P* < 0.0001; 80 μg/ml: *F*_(5, 48)_ = 83.54, *P* < 0.0001), and IFN-γ (0 μg/ml: *F*_(5, 48)_ = 83.54, *P* = 0.0839; 10 μg/ml: *F*_(5, 48)_ = 83.54, *P* < 0.0001; 20 μg/ml: *F*_(5, 48)_ = 83.54, *P* < 0.0001; 40 μg/ml: *F*_(5, 48)_ = 83.54, *P* < 0.0001; 80 μg/ml: *F*_(5, 48)_ = 83.54, *P* < 0.0001).

### rFgRab10 protein promotes goat PBMCs NO production

**Fig. 4:**
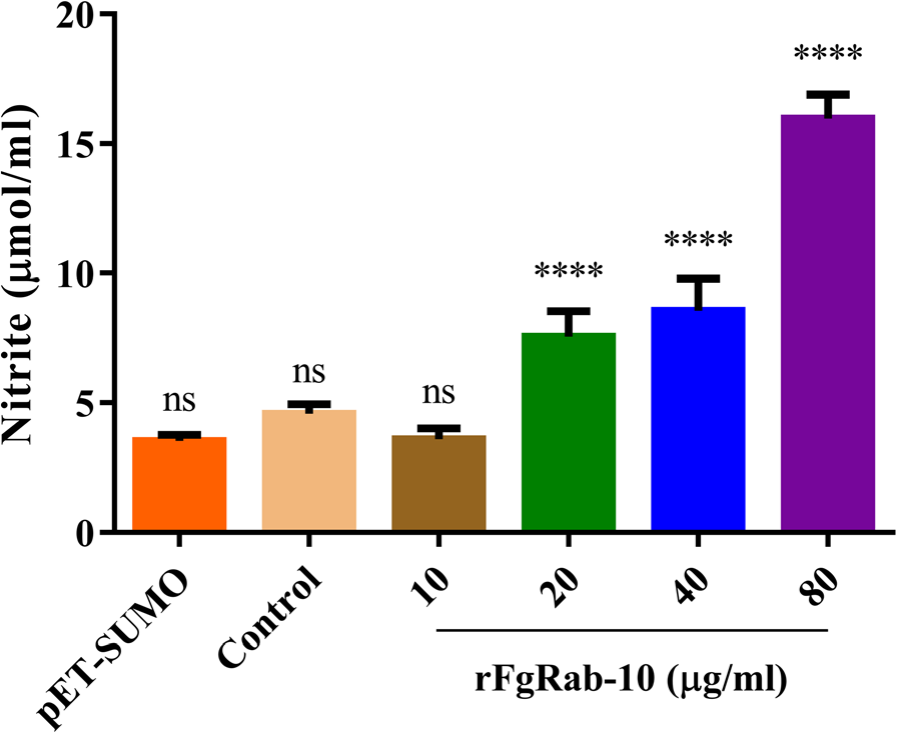
Effects of rFgRab10 protein on the production of intracellular NO. PBMCs were sham-treated with PBS, SUMO protein or serial concentrations of rFgRab10 protein and incubated for 24 h at 37 °C at 5% CO_2_. NO concentration in the PBMCs was measured using Griess assay. Graphs represent means ± SD of data from 3 independent biological replicates. Asterisks indicate statistical significance between the different indicated groups (****P < 0.0001; ns, non-significant)

As shown in Fig. 4, NO release from PBMCs was significantly increased in the presence of rFgRab10 protein compared with controls, but not at 0 μg/ml and 10 μg/ml (ANOVA tests: 0 μg/ml: *F*_(5, 24)_ = 126.2, *P* = 0.2183; 10 μg/ml: *F*_(5, 24)_ = 126.2, *P* = 0.9998; 20 μg/ml: *F*_(5, 24)_ = 126.2, *P* < 0.0001; 40 μg/ml: *F*_(5, 24)_ = 126.2, *P* < 0.0001; 80 μg/ml: *F*_(5, 24)_ = 126.2, *P* < 0.0001).

### rFgRab10 protein stimulates goat PBMCs apoptosis

**Fig. 5:**
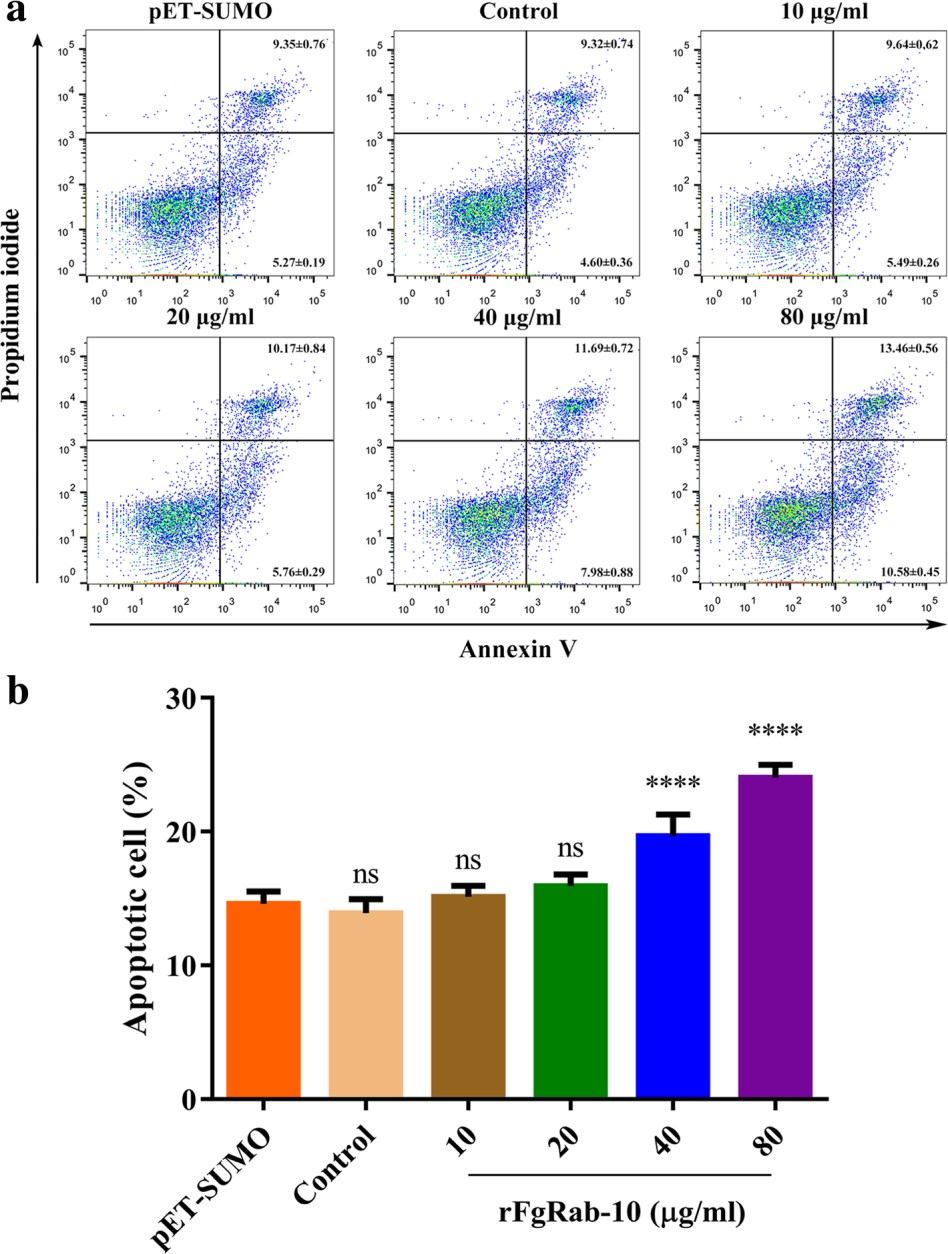
rFgRab10 protein induced apoptosis in PBMCs. Annexin V/PI staining combined with flow cytometry analysis was used to count apoptotic cells. **a** Dot plots showing apoptosis of PBMCs in response to exposure to rFgRab10 protein. **b** Percentage of cell population was compared with plotted apoptotic cells (Annexin V+/PI-). Graphs represent means ± SD of data from three independent biological replicates. Asterisks indicate statistical significance between rFgRab10-treated, SUMO protein and sham-treated PBMCs (****P < 0.0001; ns, non-significant). The statistical significance was only achieved at the two highest concentrations (40 μg/ml and 80 μg/ml)

We monitored the level of apoptosis in rFgRab10-treated PBMCs using the Annexin V-FITC/PI double staining apoptosis assay. rFgRab10 protein significantly induced apoptosis in PBMCs in a dose-dependent manner (ANOVA tests: 0 μg/ml: *F*_(5, 48)_ = 117.6, *P* = 0.5249; 10 μg/ml: *F*_(5, 48)_ = 117.6, *P* = 0.7685; 20 μg/ml: *F*_(5, 48)_ = 117.6, *P* = 0.0531; 40 μg/ml: *F*_(5, 48)_ = 117.6, *P* < 0.0001; 80 μg/ml: *F*_(5, 48)_ = 117.6, *P* < 0.0001) (Fig. 5).

### rFgRab10 protein stimulates goat PBMCs migration

**Fig. 6:**
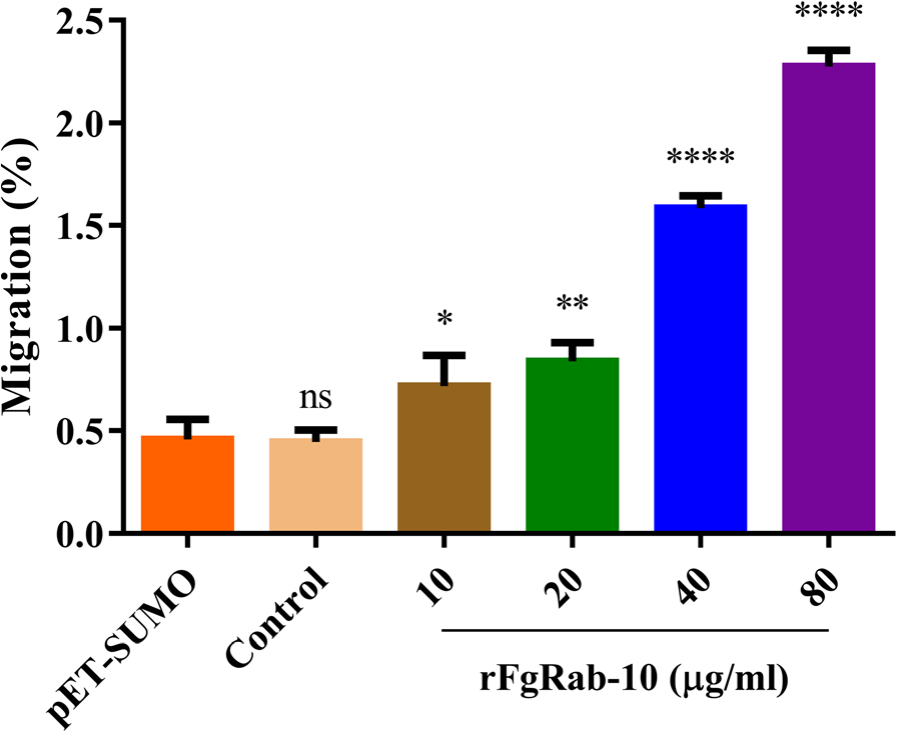
rFgRab10 protein promoted goat PBMCs migration. PBMCs were treated with PBS, SUMO protein or serial concentrations of rFgRab10 protein and then the goat PBMCs migration percentage (%) was determined. Graphs represent means ± SD of data from 3 independent biological replicates. Asterisks indicate statistical significance between the different indicated groups (*P < 0.05; **P < 0.01; ****P < 0.0001; ns, non-significant)

rFg14-3-3e protein significantly promoted, in a dose-dependent manner, the migration of goat PBMCs at all tested concentrations compared to control PBMCs treated with SUMO protein (10 μg/ml: *F*_(5, 12)_ = 176.5, *P* = 0.0230; 20 μg/ml: *F*_(5, 12)_ = 176.5, *P* = 0.0015; 40 μg/ml: *F*_(5, 12)_ = 176.5, *P* < 0.0001; 80 μg/ml: *F*_(5, 12)_ = 176.5, *P* < 0.0001; one-way ANOVA) (Fig. 6).

### rFgRab10 protein increases goat monocyte phagocytosis

**Fig. 7:**
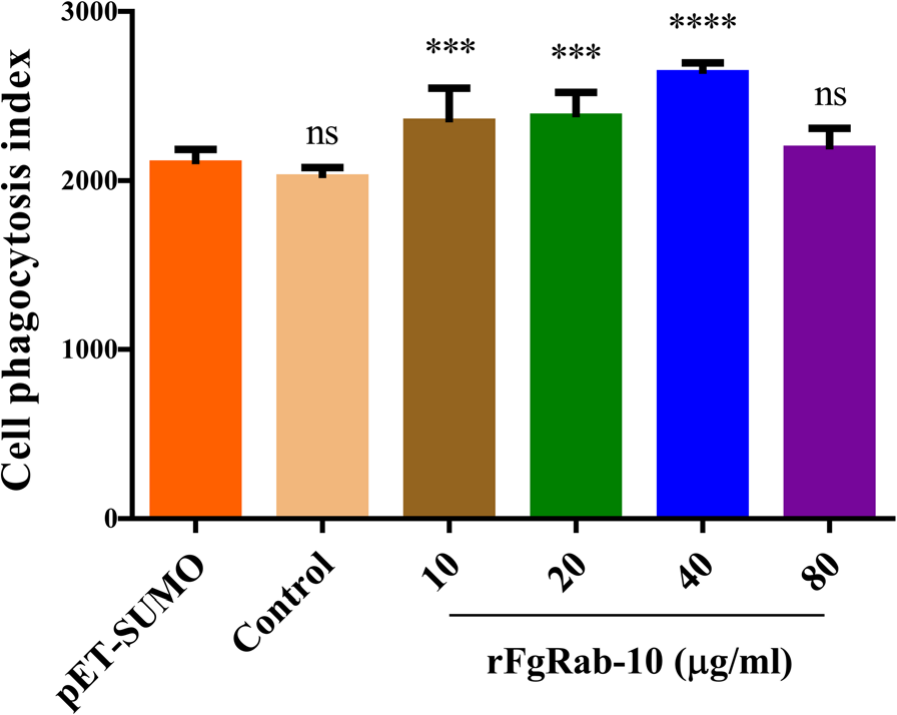
rFgRab10 protein promoted phagocytosis of goat monocytes. rFgRab10 protein increased the phagocytic ability of goat monocytes as indicated by the increase in FITC-dextran uptake in goat monocytes treated with serial concentrations of rFgRab10 protein, compared with those treated with PBS or SUMO protein. Graphs represent means ± SD of data from three independent biological replicates. Asterisks indicate statistical significance between the different indicated groups (***P < 0.001; ****P < 0.0001; ns, non-significant)

We determined whether rFgRab10 protein influences the phagocytic ability of monocytes that were cultured with PBS, SUMO protein or serial concentrations of rFgRab10 protein prior to exposure to FITC-dextran. The phagocytic capability of monocytes was examined by assessment of FITC-dextran uptake. rFgRab10 protein at 10 μg/ml, 20 μg/ml and 40 μg/ml (but not at 80 μg/ml) significantly increased the phagocytic ability of monocytes compared with cells incubated with SUMO protein (10 μg/ml: *F*_(5, 48)_ = 28.37, *P* = 0.0006; 20 μg/ml: *F*_(5, 48_) = 28.37, *P* < 0.0001; 40 μg/ml: *F*_(5, 48)_ = 28.37, *P* < 0.0001; *F*_(5, 48)_ = F7 495 28.37, *P* = 0.4258; oneway ANOVA) (Fig. 7).

### rFgRab10 protein inhibits goat PBMCs proliferation

**Fig. 8:**
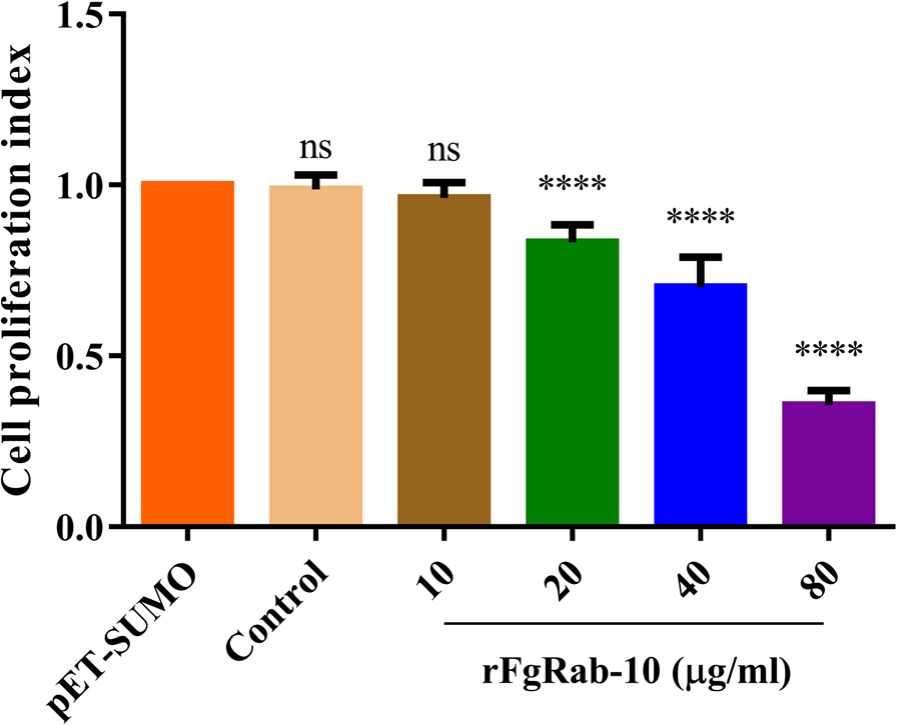
rFgRab10 protein inhibited goat PBMCs proliferation. Goat PBMCs were treated with PBS, SUMO protein or serial concentrations of rFgRab10 protein and incubated for 48 h at 37 °C at 5% CO_2_. Proliferation of goat PBMCs was determined using CCK-8 assay. Results indicated that rFgRab10 protein significantly inhibited PBMCs proliferation. Mean values and the respective standard deviation of three independent experiments are presented. Asterisks indicate statistical significance between the different indicated groups (****P < 0.0001; ns, non-significant)

rFgRab10 protein at 20 μg/ml, 40 μg/ml and 80 μg/ml concentrations (but not at 10 μg/ml) significantly inhibited PBMCs proliferation compared with PBMCs treated with SUMO protein obtained from *E. coli* transformed with empty pET-SUMO vector (10 μg/ml: *F*_(5, 30)_ = 140.5, *P* = 0.5720; 20 μg/ml: *F*_(5, 30)_ = 140.5, *P* < 0.0001; 40 μg/ml: *F*_(5, 30)_ = 140.5, *P* < 0.0001; 80 μg/ml: *F*_(5, 30)_ = 140.5, *P* < 0.0001; one-way ANOVA) (Fig. 8).

### 3D modeling

**Fig. 9:**
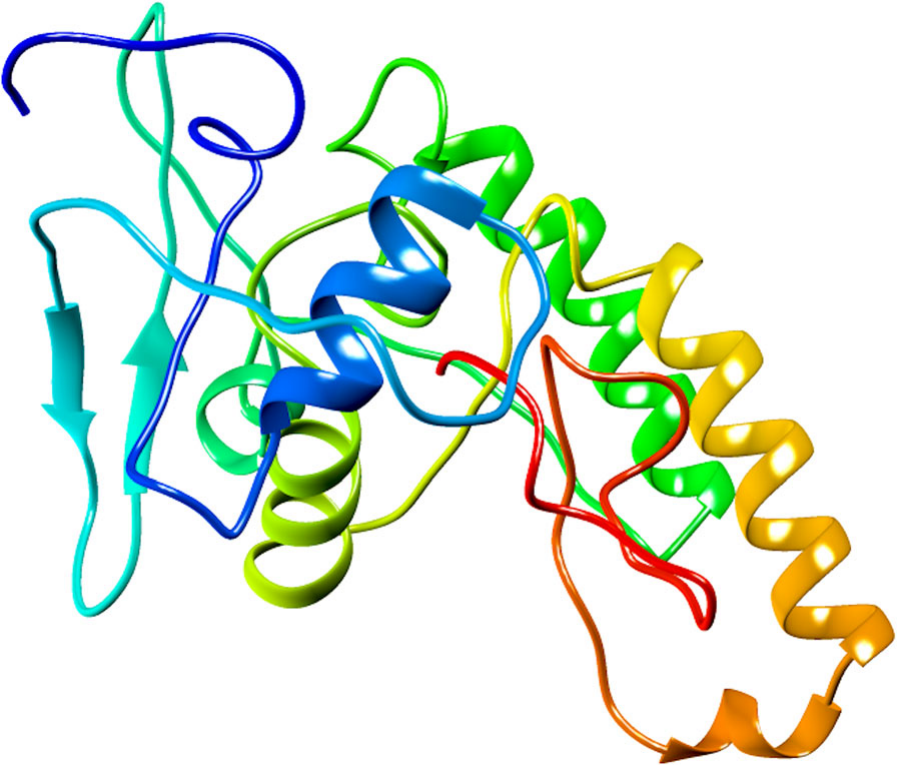
*De novo* 3D *ab initio* model for rFgRab10 protein. The model was generated using Rosetta

**Fig. 10:**
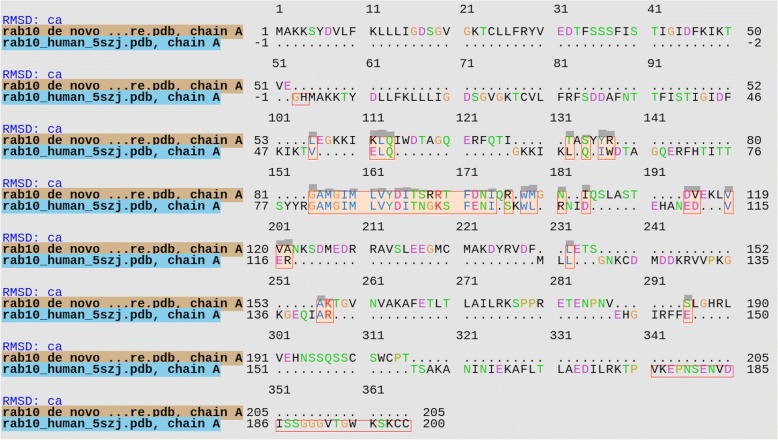
The structure alignment of rFgRab10 to the human Rab10 protein. The predicted rFgRab10 model shared 63% similarity with the human Rab10 protein (Uniprot ID: P61026)

**Fig. 11:**
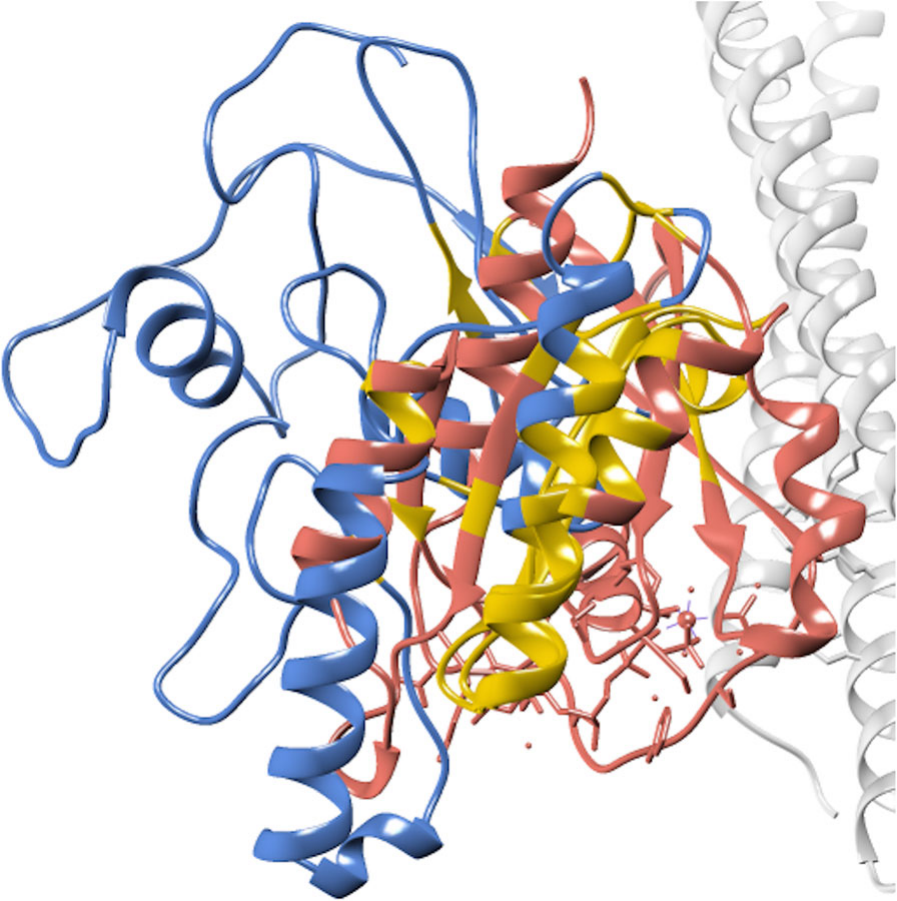
3D representation of both rFgRab10 protein and human Rab10 protein: Chain A of human Rab10 protein (red) and rFgRab10 protein (blue), with the aligned region shown in yellow

The Protein Blast result showed that the rFgRab10 protein was very similar to Chain A, structure of Human Rab10, with an identity of 63%, an E-value of 3e-85, a Query cover of 90% and a Max score of 253. The human Rab10 protein is a 200 amino-acid protein, with a molecular mass of ~22.5 KDa and is annotated as Ras-related protein Rab10 (Uniprot ID: P61026). *De novo* rFgRab10 protein structure (Fig. 9) was similar to human Rab10 protein. Figure 10 shows the structure alignment of rFgRab10 protein to the human Rab10 protein, and Fig. 11 shows a 3D representation of both proteins at the same time: Chain A of Rab10 human protein (red) and rFgRab10 protein (blue), with the aligned region shown in yellow.

### Protein interactions analysis

**Fig. 12:**
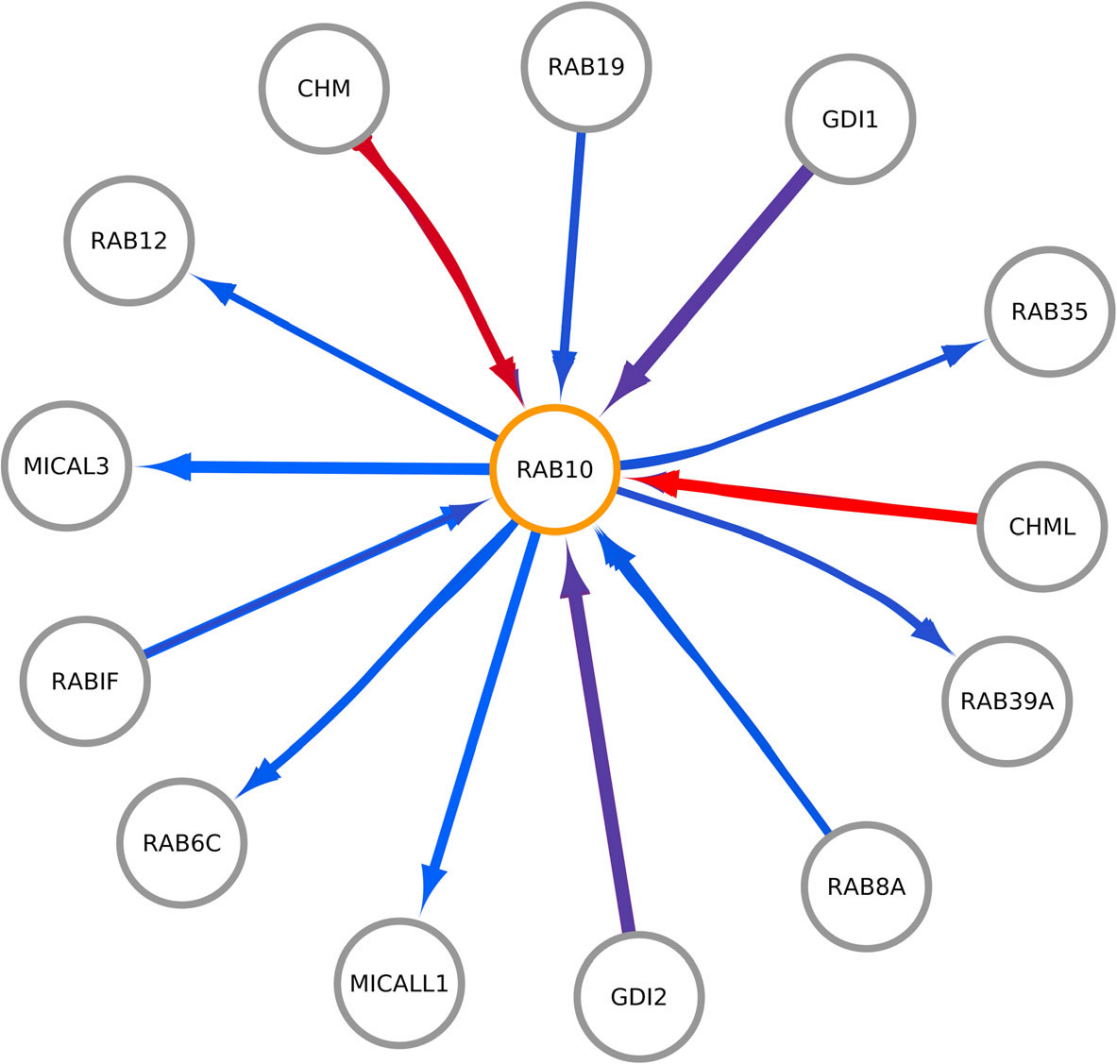
The protein interaction network revealed more stable interactions, compared to the human ortholog Rab10, between Rab proteins geranylgeranyltransferase component A 1 (CHM) and Rab proteins geranylgeranyltransferase component A 2 (CHML) and rFgRab10 sequence (red edges); whereas fewer stable interactions were detected between [F-actin]-monooxygenase MICAL3 (MICAL3) and Guanine nucleotide exchange factor MSS4 (RABIF) proteins and rFgRab10 (blue edges)

Interactome3D analysis detected 36 interactions between human Rab10 (P61026) and 13 other human proteins. All interactions with their respective total energy for the WildType (P61026 - human Rab10), Total Energy Mutant (rFgRab10 protein sequence aligned to P61026), and the difference of the previous values are provided in Additional file 1: Table S1. The protein interactions network (Fig. 12) showed that Rab proteins geranylgeranyltransferase component A 1 (CHM) and Rab proteins geranylgeranyltransferase component A 2 (CHML) had more stable interactions (red edges) with rFgRab10 protein compared to the human ortholog, whereas [F-actin]-monooxygenase MICAL3 (MICAL3) and guanine nucleotide exchange factor MSS4 (RABIF) had fewer stable interactions (blue edges) with rFgRab10 protein. These interacting partners were enriched in terms related to RabGTPase signalling, with Rab GDP-dissociation inhibitor activity (GO:0005093), Rab-protein geranylgeranyltransferase complex (GO:0005968) and protein geranylgeranylation (GO:0018344), being the overrepresented GO terms (Additional file 2: Table S2).

## Discussion

To establish a persistent infection in their mammalian definitive host, liver flukes have developed sophisticated strategies to interfere with the host’s immune response. In a recent study, we showed that rFg14-3-3e protein, another *F. gigantica* ESP, modulates several cellular and immunological functions of goat PBMCs [6]. In the present study, the gene encoding Rab10 of *F. gigantica* was amplified by PCR, cloned, and expressed in a prokaryotic expression system. SDS-PAGE and Western blot analysis of the recombinant protein (rFgRab10) using sera from *Fasciola*-infected sheep revealed a molecular mass of ~23 kDa. rFgRab10 protein cross-reacted with anti-rFgRab10 antibody as indicated by specific binding of rFgRab10 protein to the surface of the PBMCs. Thus, it is reasonable to hypothesize that stimulation of goat PBMCs with rFgRab10 protein may interfere with several functions of PBMCs. Understanding the mechanisms of rFgRab10 interaction with PBMCs has important *in vivo* relevance for the definition of early events of the cellular responses to rFgRab10 released by liver flukes during infection. Therefore, we examined the influences of rFgRab10 protein on various functions of goat PBMCs. When incubated with PBMCs, rFgRab10 protein significantly (i) promoted the secretion of IL-2, IL-4, IL-10, TGF-β, and IFN-γ; (ii) increased the release of total NO from PBMCs; (iii) stimulated goat PBMCs apoptosis and migration; and (iv) enhanced monocyte phagocytosis; but (v) inhibited PBMCs proliferation. Alterations of these functions in PBMCs may contribute to multiple aspects of *F. gigantica* pathogenesis.

Cytokines are produced by a variety of leukocytes and play many roles in maintaining immune homeostasis in the host [25, 26]. *Fasciola gigantica* infection can induce a mixed Th1/Th2 immune response [27, 28]. Our evaluation of the cytokine production [Th1 (IFN-γ and IL-2) and Th2 (IL-4, IL-10 and TGF-β)] in PBMCs culture supernatants showed that rFgRab10 protein can induce both humoral and cellular immune responses, confirming an earlier observation [27, 28]. Th1 cells secrete IFN-γ, which activates monocytes, thereby promoting the production of NO and phagocytosis [29], which may influence the development of the adaptive cellular immune response. Th2 cells secrete IL-4 to mediate humoral immunity and control helminthic infection [30] *via* the production of IgE by B cells, which can activate antibody-dependent killing of eosinophils, macrophages and mast cells [31–33]. Earlier studies indicated that apoptotic cells may participate in suppressing inflammatory responses through inducing the anti-inflammatory cytokines IL-10 [34, 35] and TGF-β [36, 37], which inhibit T-cell activation and differentiation [38]. Thus, reduced proliferation of rFgRab10-treated PBMCs is likely to be caused by cytokines IL-10 and TGF-β, released from apoptotic PBMCs.

Induction of apoptosis to prevent potentially harmful immune responses has been reported in macrophages and eosinophils exposed to *F. hepatica* ESPs [39, 40]. The same observation has been reported in peritoneal leucocytes of sheep experimentally infected with *F. hepatica*, and has been assumed to facilitate the juvenile flukes survival during the migration through the peritoneum to the liver [41]. *Fasciola hepatica* seemed to suppress the inflammatory response by inducing apoptosis in goat PBMCs [42]. Likewise, *F. gigantica* rFg14-3-3e protein significantly induced apoptosis in goat PBMCs [6]. In support of these findings, we have demonstrated the induction of apoptosis in goat PBMCs cultures following *in vitro* exposure to rFgRab10 protein. rFgRab10-mediated reduction in cellular proliferation and increased apoptosis of PBMCs seems to be mechanisms employed by *F. gigantica* to evade host immune defenses.

A previous study indicated that the intrinsic mitochondrial pathway and the extrinsic death receptor pathway may impact *F. hepatica*-induced apoptosis in goat PBMCs [42]. However, apoptotic mechanisms may vary in response to different parasite effectors and the mechanism that underpins rFgRab10 protein-induced apoptosis remains unknown. The host immune system produces substances with oxidizing activities, such as NO, reactive oxygen species (ROS) and reactive nitrogen intermediates (RNI) in order to counter the parasite infection [43, 44]. NO can be released by a variety of immune cells, especially in response to IFN-γ stimulation [29, 45]. Our data show that rFgRab10 protein promoted the secretion of IFN-γ from PBMCs [46]. IFN-γ is a potent inducer of nitric oxide synthase 2 (Nos2) in a range of leukocytes, which converts l-arginine into l-citrulline and NO [47]. NO may therefore serve as a mediator of IFN-γ-induced cellular damage. Further studies are warranted to investigate the process by which rFgRab10 protein induces apoptosis in goat PBMCs.

Our bioinformatics analysis revealed 63% identity between rFgRab10 and human Rab10 protein (Uniprot ID: P61026). The diverse roles of rFgRab10 protein suggest that this protein functions through coordinated interactions with several regulatory partners. Thus, we used human Rab10 as an input to identify potential interactions of rFgRab10 protein with human proteins in major databases. Interactions identified between rFgRab10 and human proteins were of special interest. The protein interaction network revealed that Rab proteins geranylgeranyltransferase component A 1 (CHM) and Rab proteins geranylgeranyltransferase component A 2 (CHML) had more stable interactions with rFgRab10 protein, whereas [F-actin]-monooxygenase MICAL3 (MICAL3) and guanine nucleotide exchange factor MSS4 (RABIF) had fewer stable interactions with rFgRab10 protein. Host-parasite protein interactions are critical determinants in pathogenesis. Our study provides the first identification of host protein partners for rFgRab10 protein. Further understanding of the interactions existing between rFgRab10 and host proteins, and how the involved proteins interact, can advance our understanding of molecular mechanisms involved in *F. gigantica* infection and provide new opportunities for developing immunomodulatory therapies. We also performed GO enrichment analysis, which identified terms related to Rab GTPase signaling, with Rab GDP-dissociation inhibitor activity, Rab-protein geranylgeranyltransferase complex, and protein geranylgeranylation. These findings suggest that rFgRab10 can initiate signaling events to modulate various cellular immune functions that may have implications in *F. gigantica* pathogenesis.

## Conclusions

To our knowledge, this is the first study to characterize the rFgRab10 protein and to illustrate that this protein, when incubated with goat PBMCs, modulates the cellular immune response, and enhances the release of NO, cell apoptosis, cell migration, and monocyte phagocytosis, but inhibits cell proliferation. The demonstration of the cross-reactivity of rFgRab10 protein with serum from infected animals, its binding to and interaction with goat PBMCs, and its influence on various cellular functions suggest that rFgRab10 protein plays various roles in the immunopathogenesis of fascioliasis. The immunomodulatory roles of rFgRab10 protein and its potential to influence the host-parasite equilibrium in the context of *F. gigantica* infection makes rFgRab10 a potentially good candidate for developing an immunomodulatory therapy for *F. gigantica* infection. Our study provided a “proof of concept” for clinical evaluation of the immunomodulatory roles of rFgRab10 protein in infected goats.

## Additional files

Additional file 1: **Table S1.** List of all interactions with its respective total energy for the WildType (P61026 - Rab10), Total Energy Mutant (rFgRab10 sequence aligned to P61026) and the difference of the previous values. (XLSX 10 kb)

Additional file 2: **Table S2.** Results of the gene ontology enrichment analysis of the interacting proteins. (XLSX 12 kb)

## Abbreviations

BSA: Bovine serum albumin
cDNA: Complementary deoxyribonucleic acid
ELISA: Enzyme linked immunosorbent assay
ESPs: Excretory/secretory proteins
FBS: Fetal bovine serum
FITC: Fluorescein isothiocyanate
HRP: Horseradish peroxidase
IFA: Immunofluorescence assay
IFN-γ: Interferon gamma
IgG: Immunoglobulin G
IL-10: Interleukin 10
IL-2: Interleukin 2
IL-4: Interleukin 4
ITS: Internal transcribed spacer
LC-MS/MS: Liquid chromatography-tandem mass spectrometry
LPS: Lipopolysaccharide
MFI: Median fluorescence intensity
NO: Nitric oxide
PBMCs: Peripheral blood mononuclear cells
PBS: Phosphate-buffered solution
PI: Propidium iodide
PS: Penicillin-streptomycin
RNA: Ribonucleic acid
RT-PCR: Reverse transcription polymerase chain reaction
SD: Sprague Dawley
SD: Standard deviation
SDS-PAGE: Sodium dodecyl sulfate polyacrylamide gel electrophoresis
TBS-T: Tris-buffered saline containing 0.1% tween-20
TGF-β: Transforming growth factor beta
